# Supplementary material for: Significant Impact of Growth Medium on Itraconazole Susceptibility in Azole-Resistant Versus Wild-Type Trichophyton indotineae, rubrum, and quinckeanum Isolates
Source: Int J Mol Sci. 2025 Jul 23;26(15):7090. doi: 10.3390/ijms26157090 (PMC12346380; doi:10.3390/ijms26157090)

International Journal of
Molecular Sciences

MDPI

Supplementary Information

Significant Impact of Growth Medium on Itraconazole Susceptibility in Azole-Resistant Versus Wild-Type *Trichophyton indotineae, rubrum,* and *quinckeanum* Isolates

Luisa Krauße, Anke Burmester, Silke Uhrlaß, Mario Fabri, Pietro Nenoff, Jörg Tittelbach and Cornelia Wiegand

Table S1. Strains, GenBank Acc. No. of ITS, *Erg1,* and *Erg11B.*

Species	Coll. No.	ITS GenBank Acc. No.	ITS best Hit GenBank Acc. No.	*Erg1* GenBank Acc. No.	*Erg11B* GenBank Acc. No.	Genome Assembly
rubrum	UKJ705/21	PV768552		OR472485	PV779182	
	UKJ706/21	PV768553		OR472486	PV779183	
	DSM16111	PV768551		PV779185	PV779184	
tonsurans	UKJ186/23	PV768556				
	UKJ1163/23		OM326340			
interdigitale	DSM16110	PV768557				
	DSM4167	PV768558				
	UKJ1780/22	PV768559				
indotineae	UKJ1676/17		MN064822			GCA_023065905
	UKJ1687/17		MN064822			GCA_023065865
	UKJ1708/17		MN064822	id. MZ636379	id. MZ636375	
	UKJ392/18		MN064822			GCA_023065795
	CBS146726	MN064822		MN068042	MZ636372	
	CBS146727	MZ647518		MT700528	MZ636373	
	UKJ262/21	MZ614626		MZ636378	MZ636376	
	UKJ476/21	MZ614627		MZ636379	MZ636375	
mentagrophytes	UKJ1722/22	PV768560				
	UKJ173/19	MN886815				
benhamiae	DSM6916	AB593393				
	UKJ117/23	PV768554				
	UKJ488/23	PV768555				
erinacei	UKJ314/23		MN974537			
quinckeanum	IHEM13697	JQ407219		OQ536515	OQ536545	
	UKJ1506/20	MW353028		OQ536511	OQ536541	
audouinii	UKJ317/23		OW984547			

canis	UKJ299/23	PV768561	
	UKJ300/23	PV768562	
floccosum	UKJ316/23		OL602457

PV GenBank Acc. No. this work; Genome assembly as cited [21]. *T. indotineae* strains from 2017-2018 were collected and analyzed for ITS and *Erg1* in a previous study [10]. Strains from routine cases between 2019 and 2021 were analyzed for ITS, *Erg1,* and *Erg11B* as previously described [17] and the corresponding *T. quinckeanum* genes from both observation periods were also analyzed in an earlier study [29].

Table S2. Primers list.

Gene	Assay	Primer name	Sequence 5'3'
ACT1	qPCR	TrACT1for1	TGTTGGTGATGAGGCACAGT
		TrACT1rev1	CCATGTCATCCCAGTTTGTG
Erg11A	qPCR	TrmErg11Afor1	GGA TCC TTA TCG ATG GGA CG
		TrmErg11Arev1	GGT AGC AAC TAT CAC CGT TAG
Erg11B	qPCR	TrmErg11Bfor1	AGA CCC TCA GTA CTT CCC TG
		TrmErg11Brev1	TGT TGG CAC CCT TGC TAA CC
Erg11B type I ampl.	PCR of type I specific fragments	TrmtypeIfor1	GCA AGT TGA AGG CAC CAA AG
		TrmtypeIrev1	CCG GAG GCT TTG TAG GGT CC
Erg11B type II ampl.	PCR of type II specific fragments	TmtypeIIfor1	CAA ATA CAC CCC AAC CAT TGG G
		TmtypeIIrev1	GAC CGA ACA GTG TCG CTG TCG

Type II and I specific primers as cited [22]; ACT1 pPCR primers as cited [47].

Table S3. Itraconazole IC_{50} values determined at three time points and two temperatures using growth media RGC, RG, RP or SG.

Strain	Medium	°C	IC_{50} µg/ mL itraconazole 120 h shaken	120 h unmoved	96 h shaken	96 h unmoved	72 h shaken	72 h unmoved
UKJ 705/21	RGC	28	0.600	0.490	0.390	0.412	0.096	0.280
		34	0.248	0.358	0.217	0.335	0.197	0.417
	RG	28	0.425	0.359	0.035	0.268	0.010	0.158
		34	0.364	0.400	0.378	0.357	0.518	0.316
	SG	28	0.420	0.490	0.255	0.412	0.131	0.280
		34	0.170	0.142	0.104	0.137	0.058	0.141
UKJ 706/21	RGC	28	0.404	0.422	0.264	0.380	0.135	0.315
		34	0.306	0.302	0.299	0.318	0.249	0.298
	RG	28	0.313	0.269	0.156	0.219	0.090	0.159
		34	0.285	0.291	0.223	0.247	0.150	0.199
	SG	28	0.482	0.103	0.200	0.380	0.104	0.315
		34	0.157	0.232	0.091	0.098	0.051	0.116
DSM16111	RGC	28	0.038	0.031	0.029	0.027	0.022	0.022
		34	0.017	0.015	0.015	0.015	0.012	0.014
	RG	28	0.043	0.027	0.037	0.024	0.027	0.020

		34	0.007	0.009	0.006	0.007	0.005	0.006
	SG	28	0.010	0.011	0.008	0.010	0.005	0.008
		34	0.005	0.003	0.004	0.002	0.003	0.002
UKJ 186/23	RGC	28	0.332	0.232	0.055	0.243	0.020	0.050
		34	0.027	0.037	0.017	0.029	0.008	0.022
	RG	28	0.039	0.015	0.030	0.005	n.d.*	n.d.
		34	0.001	0.001	0.001	0.001	0.001	0.001
	SG	28	0.010	0.005	0.007	0.007	0.015	n.d.
		34	0.00006	0.0005	0.00006	n.d.	0.00010	n.d.
UKJ 1163/23	RGC	28	0.355	0.485	0.286	0.456	0.189	n.d.
		34	0.308	0.296	0.278	0.324	0.185	n.d.
	RG	28	0.133	0.130	0.124	0.143	n.d.	n.d.
		34	0.177	0.294	0.141	0.260	0.098	0.224
	SG	28	0.141	0.135	0.114	0.096	0.096	0.081
		34	0.056	0.115	0.031	0.105	0.017	0.097
DSM16110	RGC	28	0.164	0.174	0.120	0.145	0.077	0.127
		34	0.120	0.160	0.097	0.126	0.069	0.116
	RG	28	0.120	0.109	0.124	0.210	0.130	n.d.
		34	0.074	0.050	0.091	0.050	0.436	0.051
	SG	28	0.195	0.240	0.151	0.156	0.076	0.108
		34	0.155	0.142	0.127	0.137	0.099	0.141
DSM 4167	RGC	28	0.146	0.103	0.104	0.082	0.082	0.079
		34	0.078	0.060	0.058	0.055	0.047	0.052
	RG	28	0.051	0.033	0.045	0.030	0.045	0.030
		34	0.031	0.022	0.125	0.021	0.025	0.019
	SG	28	0.123	0.103	0.091	0.082	0.060	0.079
		34	0.074	0.104	0.055	0.100	0.038	0.090
UKJ 1780/22	RGC	28	0.267	0.232	0.242	0.232	0.226	0.242
		34	0.177	0.214	0.169	0.205	0.150	0.198
	RG	28	0.105	0.104	0.103	0.103	0.102	0.098
		34	0.071	0.031	0.031	0.023	0.040	0.014
	SG	28	0.184	0.124	0.127	0.147	0.074	0.122
		34	0.045	0.020	0.023	0.030	0.010	0.073
UKJ 1676/17	RGC	28	0.100	0.101	0.095	0.100	0.087	0.101
		34	0.058	0.064	0.055	0.065	0.049	0.068
	RG	28	0.037	0.028	0.035	0.027	0.031	0.029
		34	0.009	0.008	0.008	0.008	0.008	0.007
	RP	34	0.0008	0.0006	0.001	0.0004	0.002	0.00002
	SG	28	0.028	0.009	0.025	0.033	0.022	0.026
		34	0.030	0.010	0.026	0.008	0.021	0.007
UKJ 1687/17	RGC	28	0.616	0.751	0.579	0.769	0.497	0.944
		34	0.328	0.461	0.312	0.448	0.292	0.404
	RG	28	0.347	0.319	0.335	0.328	0.362	0.550
		34	0.046	0.296	0.042	0.262	0.038	0.150
	RP	34	0.088	0.052	0.080	0.053	0.088	0.023
	SG	28	0.294	0.249	0.233	0.056	0.142	0.032

		34	0.290	0.137	0.227	0.062	0.115	0.036
UKJ 1708/17	RGC	28	0.388	0.438	0.356	0.424	0.296	0.400
		34	0.314	0.386	0.299	0.393	0.268	0.377
	RG	28	0.235	0.223	0.215	0.228	0.199	0.233
		34	0.204	0.297	0.184	0.381	0.164	0.492
	RP	34	0.047	0.084	0.045	0.050	0.054	0.043
	SG	28	0.148	0.169	0.123	0.155	0.086	0.233
		34	0.164	0.076	0.227	0.077	0.168	0.084
UKJ 392/18	RGC	28	0.633	0.801	0.594	0.802	0.525	0.730
		34	0.304	0.276	0.283	0.267	0.233	0.182
	RG	28	0.892	0.944	0.894	0.867	0.526	0.897
		34	0.409	0.342	0.381	0.343	0.324	0.419
	RP	34	0.325	0.313	0.307	0.295	0.261	0.244
	SG	28	0.407	0.449	0.248	0.367	0.143	0.234
		34	0.383	0.077	0.247	0.127	0.156	0.076
CBS 146726	RGC	28	0.349	0.380	0.310	0.330	0.241	n.d.
		34	0.333	0.297	0.285	0.310	n.d.	n.d.
	RG	28	0.293	0.210	0.271	0.185	0.253	0.172
		34	0.197	0.118	0.170	0.115	0.163	0.109
	SG	28	0.032	0.027	0.025	0.035	0.034	0.054
		34	0.014	0.019	0.011	0.018	n.d.	0.017
CBS 146727	RGC	28	0.405	0.360	0.394	0.361	0.362	n.d.
		34	0.284	0.286	0.282	n.d.	0.270	n.d.
	RG	28	0.312	0.208	0.331	0.217	0.358	0.398
		34	0.087	0.128	0.099	0.135	0.114	0.149
	SG	28	0.030	0.020	0.030	0.019	0.023	0.016
		34	0.013	0.019	0.012	0.018	0.010	0.018
UKJ 262/21	RGC	28	0.173	0.100	0.124	0.067	0.079	0.130
		34	0.149	0.131	0.120	0.116	0.091	0.101
	RG	28	0.180	0.149	0.166	0.137	0.143	0.123
		34	0.139	0.090	0.125	0.086	0.110	0.076
	SG	28	0.074	0.084	0.059	0.085	0.038	0.081
		34	0.029	0.038	0.020	0.033	0.012	0.026
UKJ 476/21	RGC	28	0.319	0.313	0.260	0.260	0.145	0.222
		34	0.370	0.304	0.257	0.292	0.183	0.268
	RG	28	0.290	0.210	0.143	0.197	n.d.	n.d.
		34	0.173	0.146	0.166	0.145	0.153	0.139
	SG	28	0.154	0.153	0.117	0.126	0.070	0.112
		34	0.064	0.104	0.041	0.100	0.026	0.090
UKJ 1722/22	RGC	28	0.305	0.503	0.301	0.481	0.265	n.d.
		34	0.178	0.210	0.165	0.200	0.141	0.230
	RG	28	0.345	0.287	0.347	0.284	0.355	0.272
		34	0.102	0.082	0.048	0.070	0.078	0.051
	SG	28	0.095	0.069	0.071	0.070	0.044	0.076
		34	0.026	0.019	0.013	0.033	n.d.	n.d.
UKJ 173/19	RGC	28	0.295	0.800	0.425	0.770	0.353	0.753

		34	0.460	0.532	0.287	0.540	0.267	0.560
	RG	28	0.334	0.316	0.348	0.313	0.367	0.304
		34	0.148	0.130	0.155	0.130	0.152	0.119
	SG	28	0.133	0.157	0.080	0.044	0.051	0.098
		34	0.039	0.052	0.016	0.064	n.d.	0.042
UKJ 117/23	RGC	28	0.669	0.684	0.656	0.612	0.599	0.514
		34	0.572	0.666	0.565	0.632	0.505	0.571
	RG	28	0.211	0.136	0.185	0.066	n.d.	n.d.
		34	0.226	0.235	0.146	0.184	0.149	0.118
	SG	28	0.095	0.152	0.065	0.150	0.040	n.d.
		34	0.042	0.054	0.026	0.045	0.015	0.039
UKJ 488/23	RGC	28	0.642	1.076	0.628	1.004	0.627	0.852
		34	1.387	1.093	0.978	1.009	0.669	0.796
	RG	28	0.342	0.297	0.373	0.204	n.d.	n.d.
		34	0.295	0.442	0.247	0.350	0.193	0.259
	SG	28	0.127	0.112	0.095	n.d.	0.066	n.d.
		34	0.076	0.111	0.050	0.086	0.024	0.061
DSM 6916	RGC	28	0.621	0.457	0.574	0.366	0.323	n.d.
		34	0.542	0.636	0.523	0.606	0.338	0.501
	RG	28	0.560	0.643	0.514	0.617	0.409	0.409
		34	0.775	0.790	1.150	1.040	0.830	1.035
	SG	28	0.519	0.850	0.283	0.630	0.149	n.d.
		34	0.122	0.551	0.122	0.193	0.042	0.075
UKJ 314/23	RGC	28	0.580	0.690	0.530	0.773	0.460	0.961
		34	0.576	0.524	0.527	0.352	0.564	0.237
	RG	28	0.299	0.499	0.294	0.454	0.248	n.d.
		34	0.281	0.533	0.489	0.490	0.218	0.440
	SG	28	0.139	n.d.	0.100	0.014	0.073	n.d.
		34	0.079	0.107	0.057	0.076	0.051	0.061
IHEM13697	RGC	28	0.257	0.269	0.231	0.261	0.182	0.243
		34	0.059	0.056	0.054	0.055	0.047	0.034
	RG	28	0.254	0.230	0.255	0.221	0.227	0.208
		34	0.210	0.049	0.190	0.048	0.163	0.049
	SG	28	0.026	0.033	0.019	0.035	0.013	0.030
		34	0.009	0.012	0.006	0.010	0.003	0.008
UKJ 1506/20	RGC	28	0.892	0.675	0.653	0.654	0.478	0.650
		34	0.392	0.642	0.330	0.623	0.284	n.d.
	RG	28	0.399	0.519	0.343	0.436	0.264	0.476
		34	0.290	0.529	0.230	0.525	0.166	n.d.
	SG	28	0.390	0.390	0.170	0.222	0.098	0.187
		34	0.190	0.230	0.100	0.170	0.051	0.216
UKJ 317/23	RGC	28	0.049	0.070	0.020	0.019	0.009	0.019
		34	0.041	0.056	0.020	0.056	n.d.	n.d.
	RG	28	n.g.#	n.g.	n.g.	n.g.	n.g.	n.g.
		34	n.g.	n.g.	n.g.	n.g.	n.g.	n.g.
	SG	28	0.177	0.074	0.054	0.025	0.024	0.012

		34	0.011	0.036	0.008	0.023	n.d.	0.017
UKJ 299/23	RGC	28	0.119	0.069	0.082	0.027	0.042	0.036
		34	0.081	0.064	0.073	0.068	0.059	0.075
	RG	28	0.035	0.060	0.035	0.058	0.050	n.d.
		34	n.g.	n.g.	n.g.	n.g.	n.g.	n.g.
	SG	28	0.034	0.102	0.019	n.d.	0.010	n.d.
		34	0.003	0.004	0.003	0.002	0.003	0.002
UKJ 300/23	RGC	28	0.046	0.041	0.029	0.033	0.015	0.033
		34	0.006	0.017	0.002	0.015	0.003	0.026
	RG	28	0.089	0.070	0.085	0.065	0.127	0.043
		34	n.g.	n.g.	n.g.	n.g.	n.g.	n.g.
	SG	28	0.009	0.011	0.006	n.d.	0.006	n.d.
		34	0.002	0.003	0.002	0.003	0.003	0.003
UKJ 316/23	RGC	28	0.095	0.079	0.057	0.040	0.033	n.d.
		34	0.125	0.095	0.077	0.081	0.037	0.049
	RG	28	0.151	0.165	0.371	0.113	n.d.	n.d.
		34	0.160	0.170	0.148	0.162	0.116	0.154
	SG	28	0.010	0.019	n.d.	n.d.	n.d.	n.d.
		34	0.009	0.009	0.008	0.007	n.d.	n.d.

* n.d.; Values were not determined due to insufficient growth at this time point. # n.g.; even the control without addition of itraconazole was not able to grow under these conditions. Continuously measured plates were shaken for 120 h, whereas unmoved plates were stored in a heat incubator and measured exclusively at time points 0 h, 1 h, 72 h, 96 h, and 120 h. Green values indicate that the time point for calculation was before the beginning of the plateau phase.

Table S4. Influence of spore titer on lag phases and their determined significance.

Medium	°C	Significance of compared spore concentration cfu/ mL on lag phase E05/E04	E05/E03	E04/03
RGC	28	<0.001	<0.001	0.003
	34	<0.001	<0.001	0.005
RG	28	0.006	<0.001	0.002
	34	0.002	<0.001	0.002
SG	28	0.002	<0.001	0.002
	34	0.001	<0.001	0.010

Figure S1. Inclusion of *T. rubrum*, *quinckeanum*, and *indotineae* sensitive and resistant isolates shown as before; highest significance for SG as growth medium.

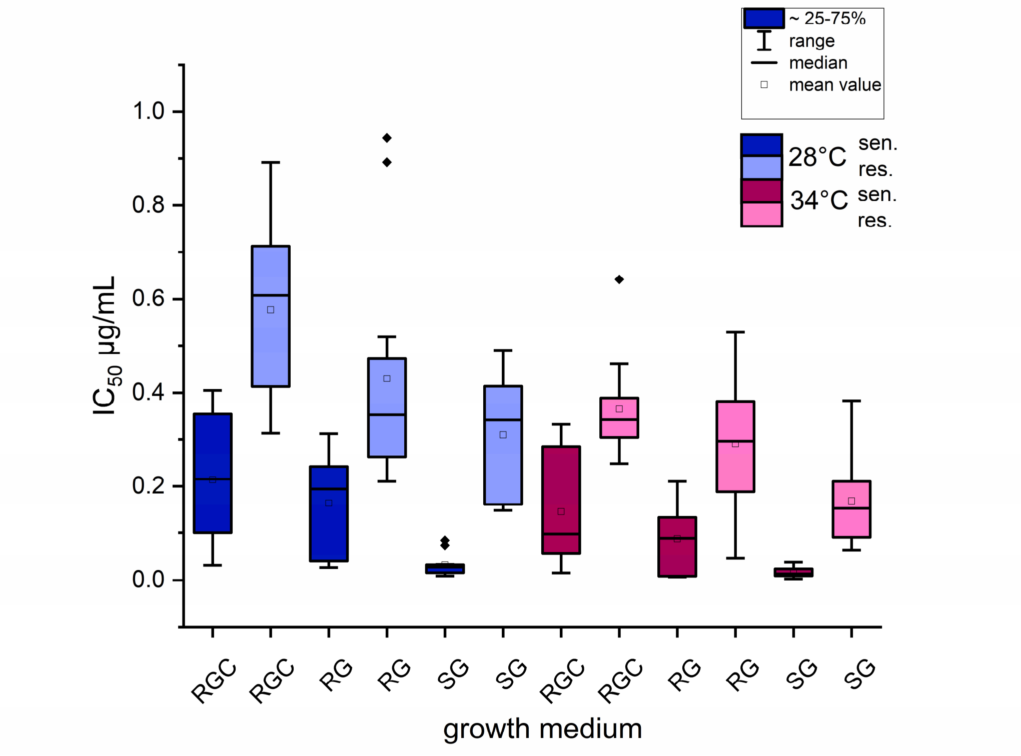

Figure S2. Media and temperature showed minimal influence depending on lag phases of isolates.

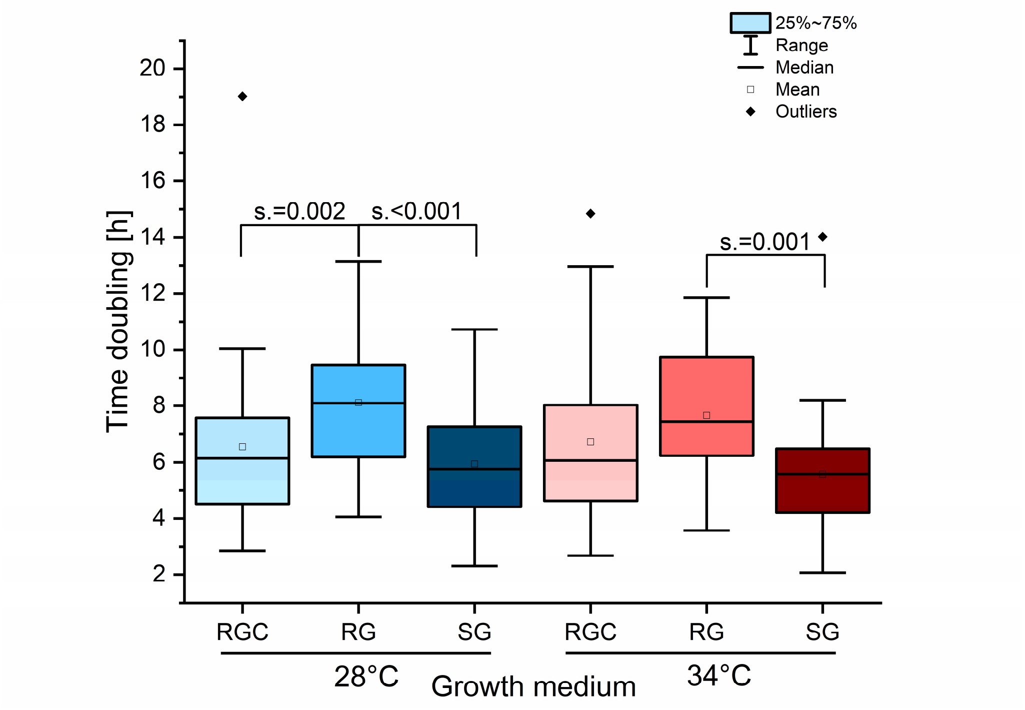

Supplement: Supplementary file 1 [file ijms-26-07090-s001.zip › ijms-3728165-supplementary.pdf]
